# Supplementary material for: Severe fever with thrombocytopenia syndrome virus induces lactylation of m6A reader protein YTHDF1 to facilitate viral replication
Source: EMBO Rep. 2024 Nov 4;25(12):5599–619. doi: 10.1038/s44319-024-00310-7 (PMC11624280; doi:10.1038/s44319-024-00310-7)
Supplement: Supplementary file 7 — Source data Fig. 1 [file 44319_2024_310_MOESM7_ESM.zip › Figure 1/1B/README.rtf]

To review GEO accession GSE256528:Go to https://www.ncbi.nlm.nih.gov/geo/query/acc.cgi?acc=GSE256528Enter token elcfseeqtpydjit into the box
