# Supplementary figures and images for: Severe fever with thrombocytopenia syndrome virus induces lactylation of m6A reader protein YTHDF1 to facilitate viral replication
Source: EMBO Rep. 2024 Nov 4;25(12):5599–619. doi: 10.1038/s44319-024-00310-7 (PMC11624280; doi:10.1038/s44319-024-00310-7)

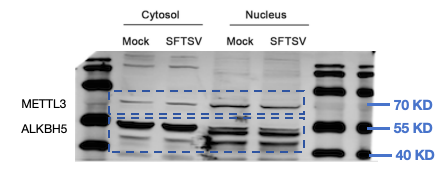

Supplement: Supplementary file 8 — Source data Fig. 2 [file 44319_2024_310_MOESM8_ESM.zip › Figure 2/2A/Wesrern blot METTL3 ALKBH5.tiff]

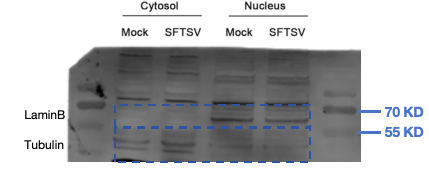

Supplement: Supplementary file 8 — Source data Fig. 2 [file 44319_2024_310_MOESM8_ESM.zip › Figure 2/2A/Wesrern blot Laminb Tubulin.tiff]

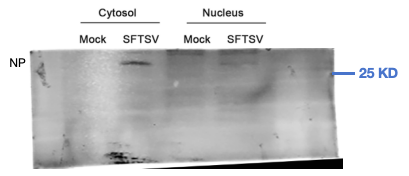

Supplement: Supplementary file 8 — Source data Fig. 2 [file 44319_2024_310_MOESM8_ESM.zip › Figure 2/2A/Wesrern blot NP.tiff]

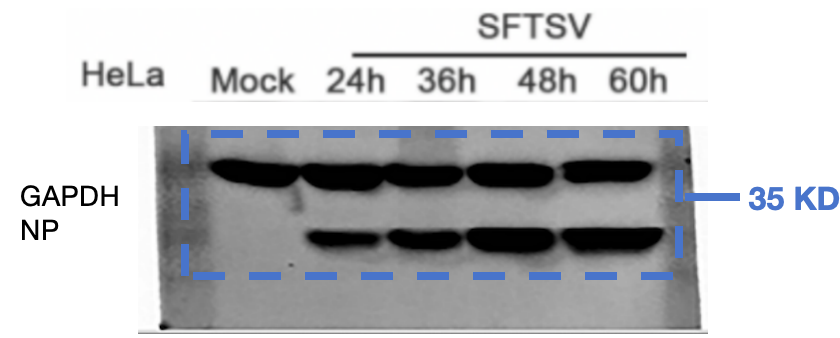

Supplement: Supplementary file 9 — Source data Fig. 3 [file 44319_2024_310_MOESM9_ESM.zip › Figure 3 /3B/Western GAPDH NP.tiff]

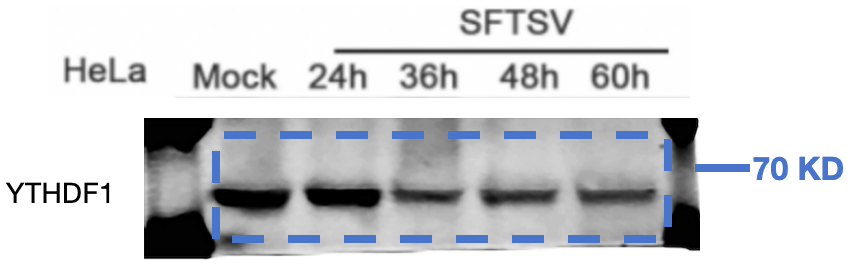

Supplement: Supplementary file 9 — Source data Fig. 3 [file 44319_2024_310_MOESM9_ESM.zip › Figure 3 /3B/WesternYTHDF1.tiff]

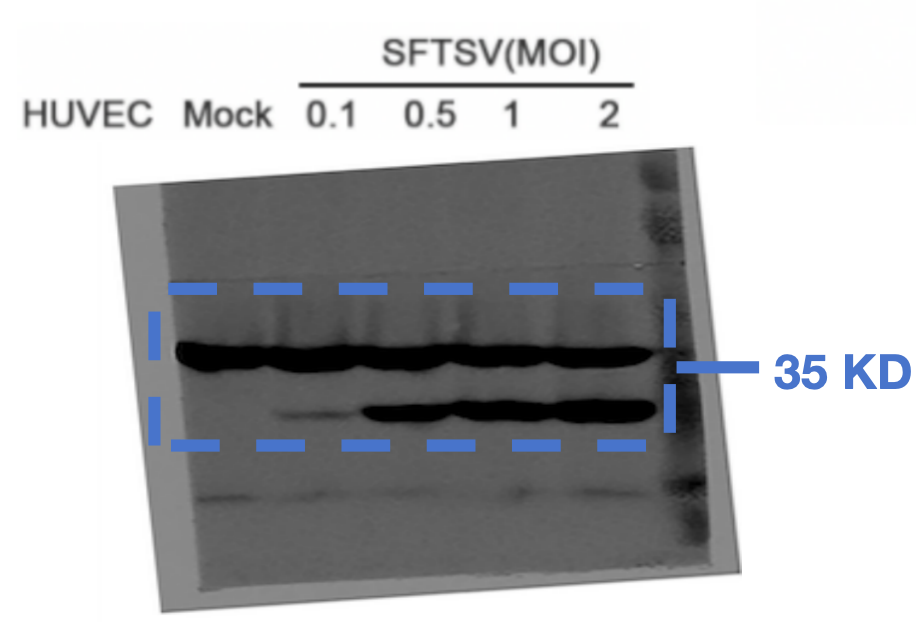

Supplement: Supplementary file 9 — Source data Fig. 3 [file 44319_2024_310_MOESM9_ESM.zip › Figure 3 /3C/Western GAPDH NP.tiff]

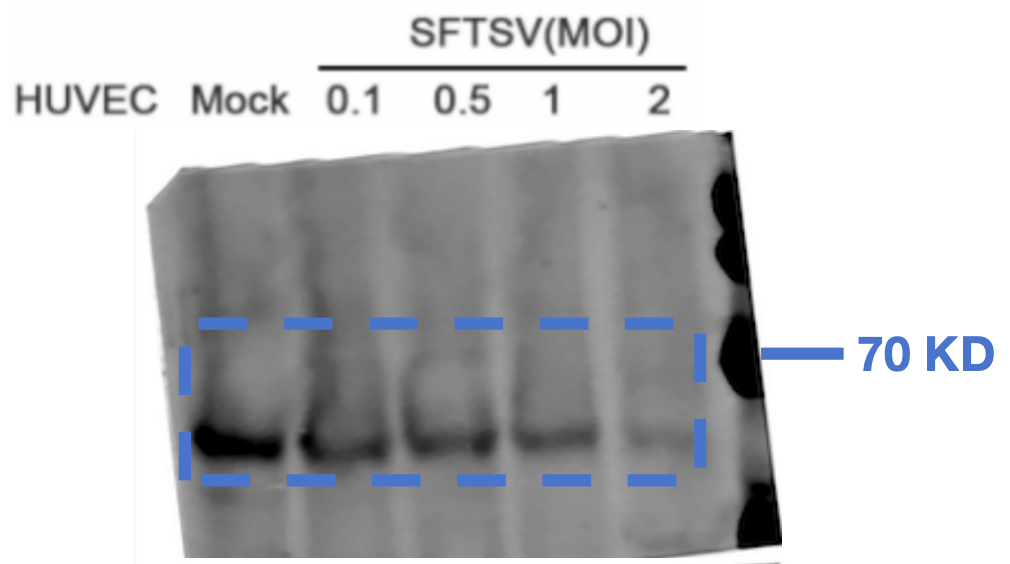

Supplement: Supplementary file 9 — Source data Fig. 3 [file 44319_2024_310_MOESM9_ESM.zip › Figure 3 /3C/Western YTHDF1.tiff]

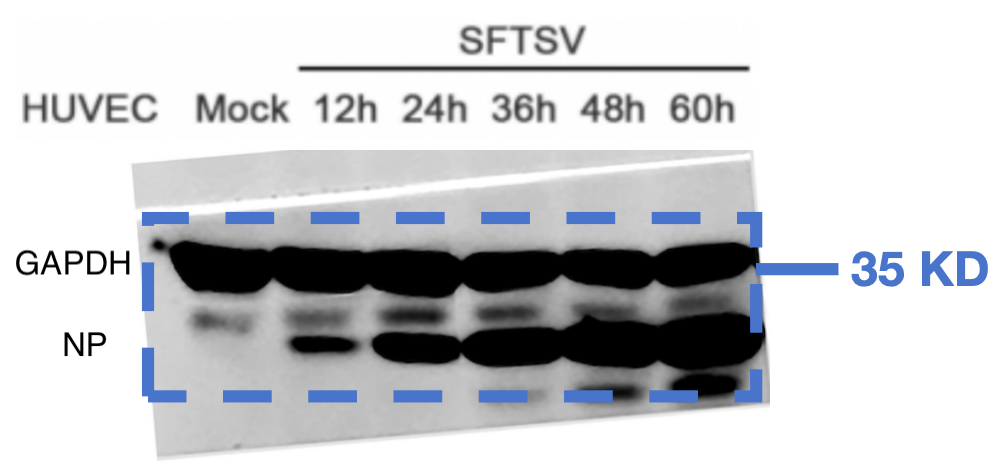

Supplement: Supplementary file 9 — Source data Fig. 3 [file 44319_2024_310_MOESM9_ESM.zip › Figure 3 /3D/Western GAPDH NP.tiff]

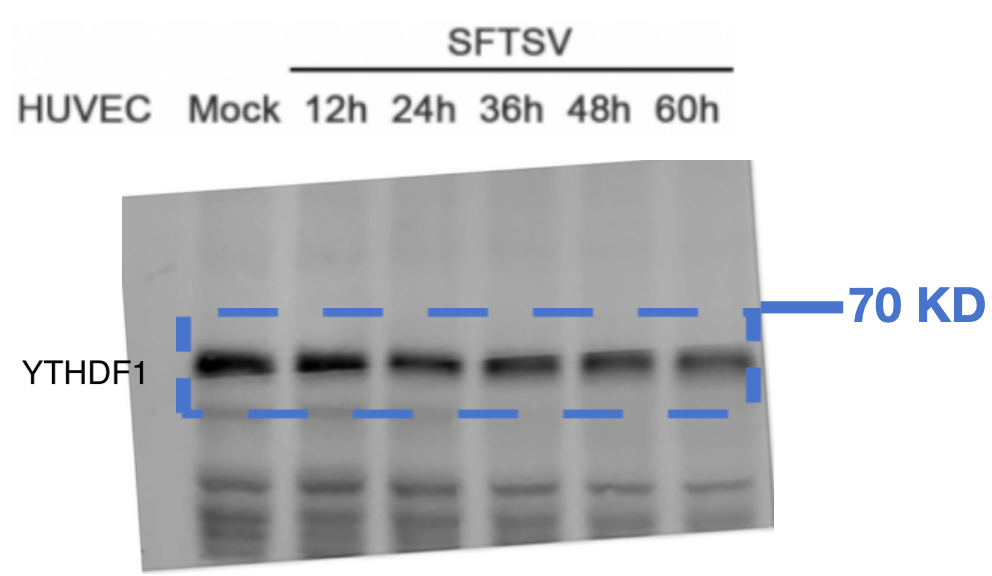

Supplement: Supplementary file 9 — Source data Fig. 3 [file 44319_2024_310_MOESM9_ESM.zip › Figure 3 /3D/Western YTHDF1.tiff]

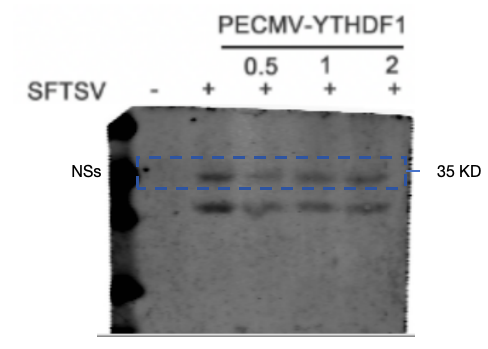

Supplement: Supplementary file 9 — Source data Fig. 3 [file 44319_2024_310_MOESM9_ESM.zip › Figure 3 /3J/Western NSs.tiff]

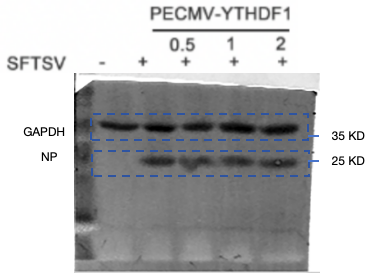

Supplement: Supplementary file 9 — Source data Fig. 3 [file 44319_2024_310_MOESM9_ESM.zip › Figure 3 /3J/Western GAPDH NP.tiff]

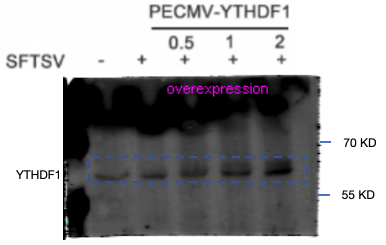

Supplement: Supplementary file 9 — Source data Fig. 3 [file 44319_2024_310_MOESM9_ESM.zip › Figure 3 /3J/Western YTHDF1.tiff]

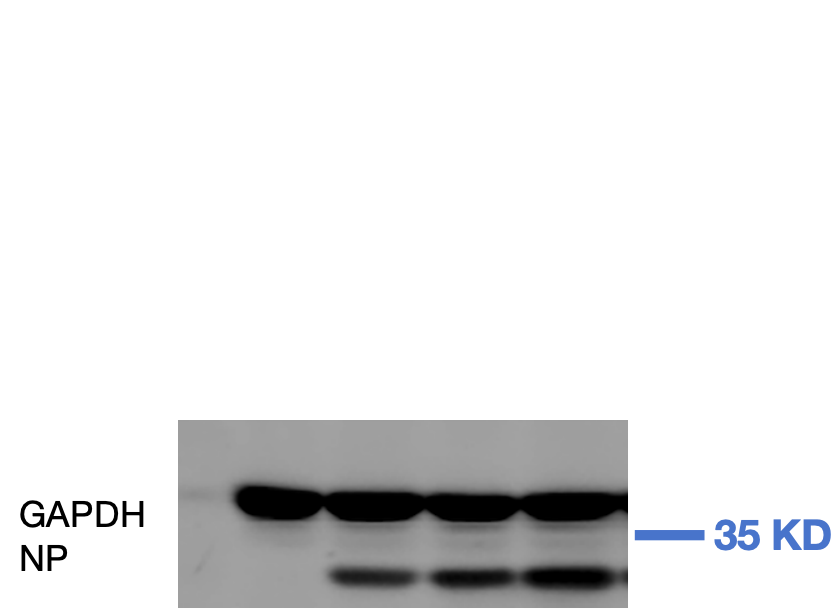

Supplement: Supplementary file 9 — Source data Fig. 3 [file 44319_2024_310_MOESM9_ESM.zip › Figure 3 /3A/western GAPDH NP.tiff]

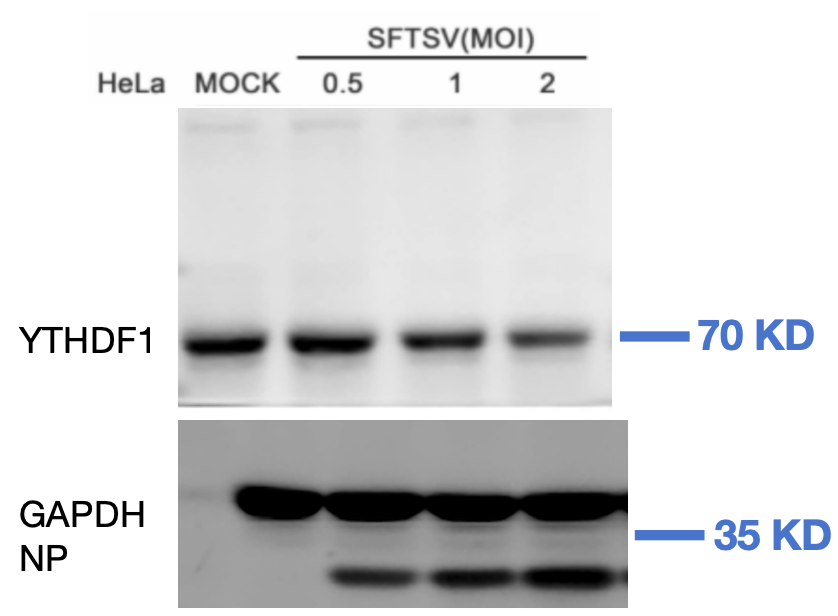

Supplement: Supplementary file 9 — Source data Fig. 3 [file 44319_2024_310_MOESM9_ESM.zip › Figure 3 /3A/western YTHDF1.tiff]

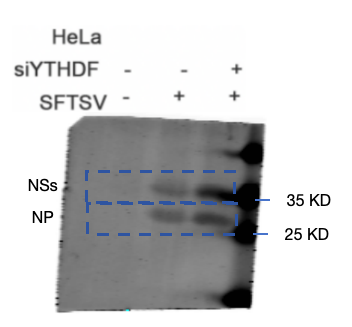

Supplement: Supplementary file 9 — Source data Fig. 3 [file 44319_2024_310_MOESM9_ESM.zip › Figure 3 /3I/Western NSs NP.tiff]

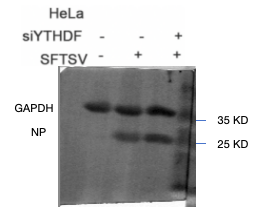

Supplement: Supplementary file 9 — Source data Fig. 3 [file 44319_2024_310_MOESM9_ESM.zip › Figure 3 /3I/Western GAPDH NP.tiff]

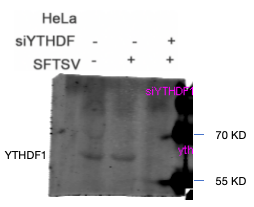

Supplement: Supplementary file 9 — Source data Fig. 3 [file 44319_2024_310_MOESM9_ESM.zip › Figure 3 /3I/Western YTHDF1.tiff]

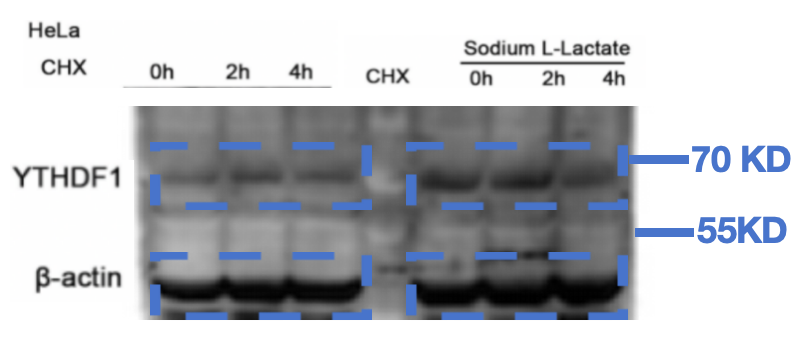

Supplement: Supplementary file 10 — Source data Fig. 4 [file 44319_2024_310_MOESM10_ESM.zip › FIgure 4/4E/Western YTHDF1 beta-actin.tiff]

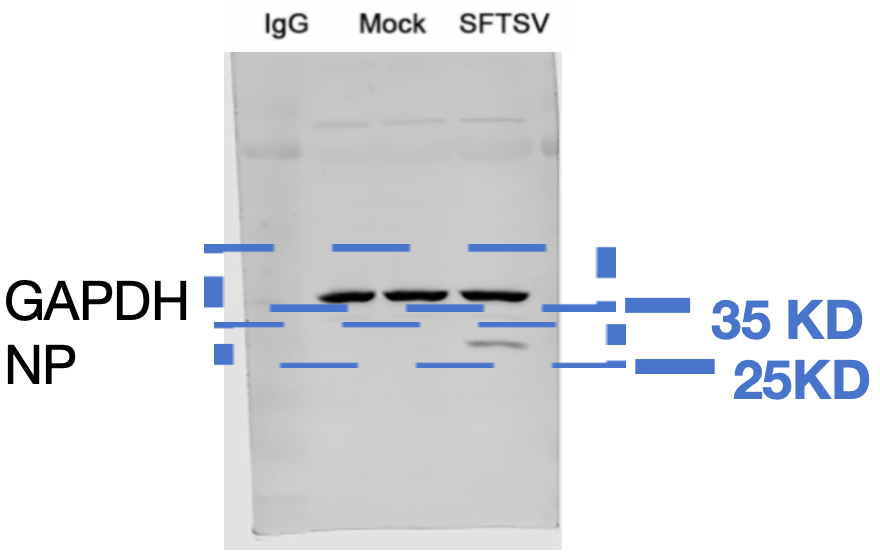

Supplement: Supplementary file 10 — Source data Fig. 4 [file 44319_2024_310_MOESM10_ESM.zip › FIgure 4/4B/Western Input_GAPDH NP.tiff]

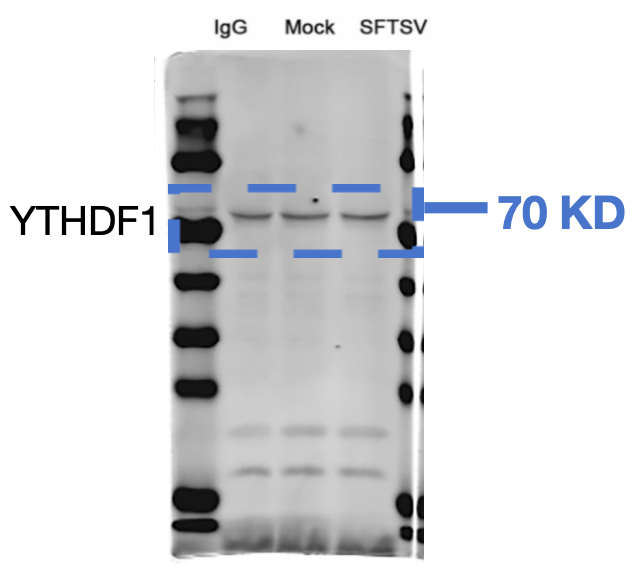

Supplement: Supplementary file 10 — Source data Fig. 4 [file 44319_2024_310_MOESM10_ESM.zip › FIgure 4/4B/Western Input_YTHDF1.tiff]

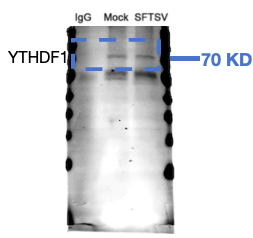

Supplement: Supplementary file 10 — Source data Fig. 4 [file 44319_2024_310_MOESM10_ESM.zip › FIgure 4/4B/Western IP YTHDF1.tiff]

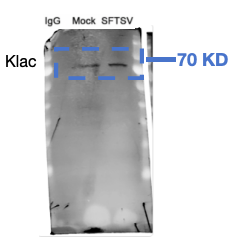

Supplement: Supplementary file 10 — Source data Fig. 4 [file 44319_2024_310_MOESM10_ESM.zip › FIgure 4/4B/Western co-IP klac.tiff]

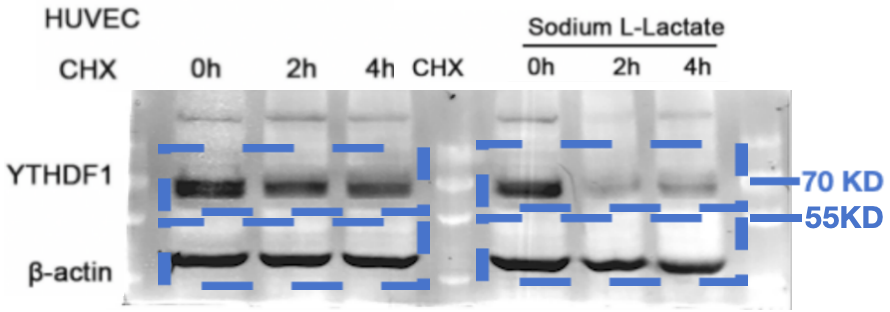

Supplement: Supplementary file 10 — Source data Fig. 4 [file 44319_2024_310_MOESM10_ESM.zip › FIgure 4/4D/Western YTHDF1 beta-actin.tiff]

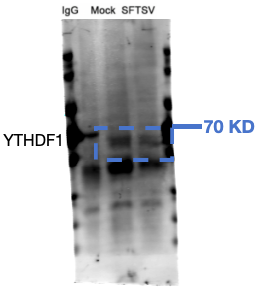

Supplement: Supplementary file 10 — Source data Fig. 4 [file 44319_2024_310_MOESM10_ESM.zip › FIgure 4/4A/Western IP YTHDF1.tiff]

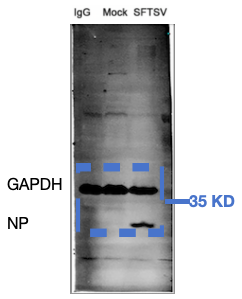

Supplement: Supplementary file 10 — Source data Fig. 4 [file 44319_2024_310_MOESM10_ESM.zip › FIgure 4/4A/Western Input GAPDH NP.tiff]

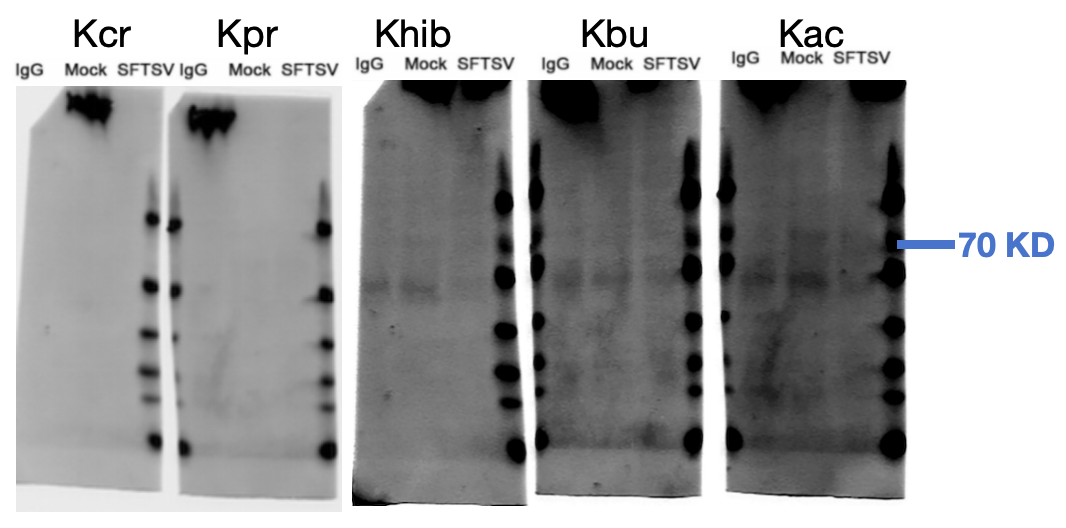

Supplement: Supplementary file 10 — Source data Fig. 4 [file 44319_2024_310_MOESM10_ESM.zip › FIgure 4/4A/Western kcr kpr khib kbu kac.tiff]

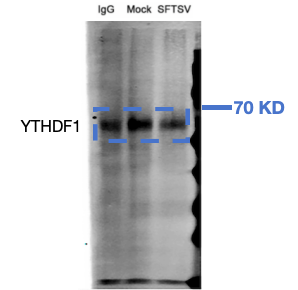

Supplement: Supplementary file 10 — Source data Fig. 4 [file 44319_2024_310_MOESM10_ESM.zip › FIgure 4/4A/Western Input YTHDF1.tiff]

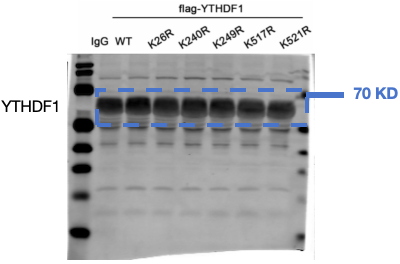

Supplement: Supplementary file 10 — Source data Fig. 4 [file 44319_2024_310_MOESM10_ESM.zip › FIgure 4/4F/Western Input_YTHDF1.tiff]

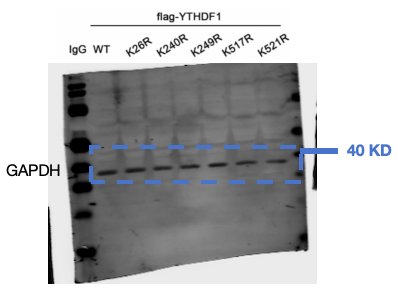

Supplement: Supplementary file 10 — Source data Fig. 4 [file 44319_2024_310_MOESM10_ESM.zip › FIgure 4/4F/Western Input_GAPDH.tiff]

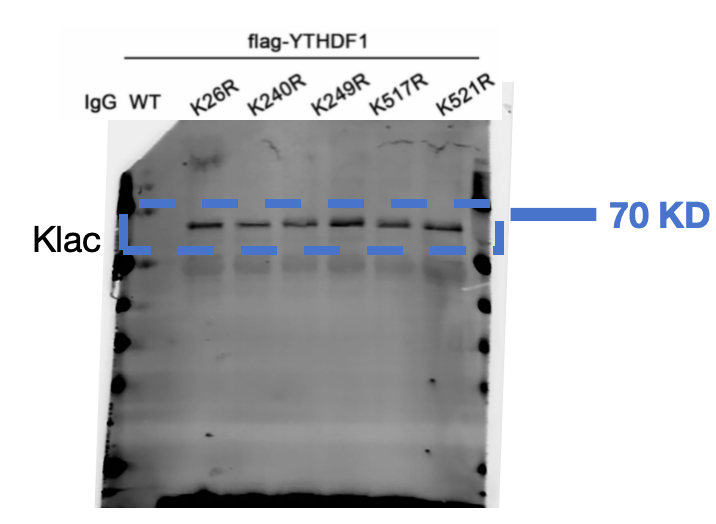

Supplement: Supplementary file 10 — Source data Fig. 4 [file 44319_2024_310_MOESM10_ESM.zip › FIgure 4/4F/Western co-IP_Klac.tiff]

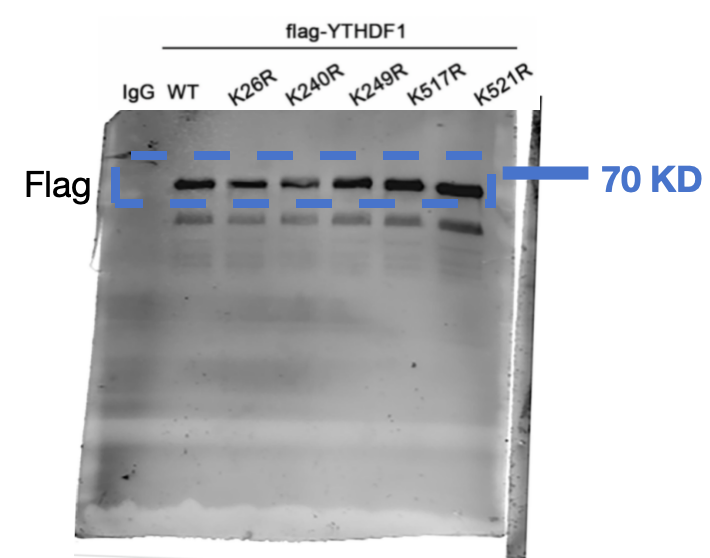

Supplement: Supplementary file 10 — Source data Fig. 4 [file 44319_2024_310_MOESM10_ESM.zip › FIgure 4/4F/Western IP_YTHDF1.tiff]

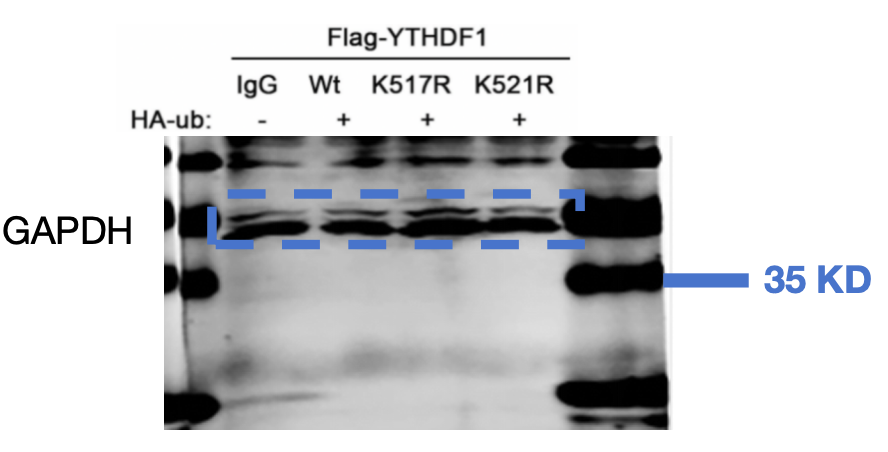

Supplement: Supplementary file 10 — Source data Fig. 4 [file 44319_2024_310_MOESM10_ESM.zip › FIgure 4/4G/Western Input_GAPDH.tiff]

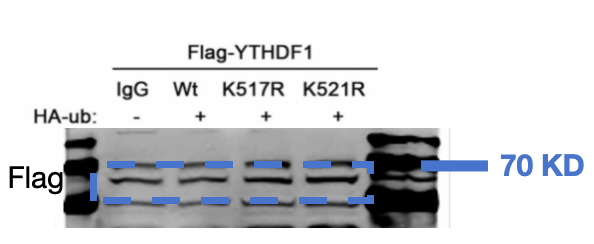

Supplement: Supplementary file 10 — Source data Fig. 4 [file 44319_2024_310_MOESM10_ESM.zip › FIgure 4/4G/Western Input_Flag.tiff]

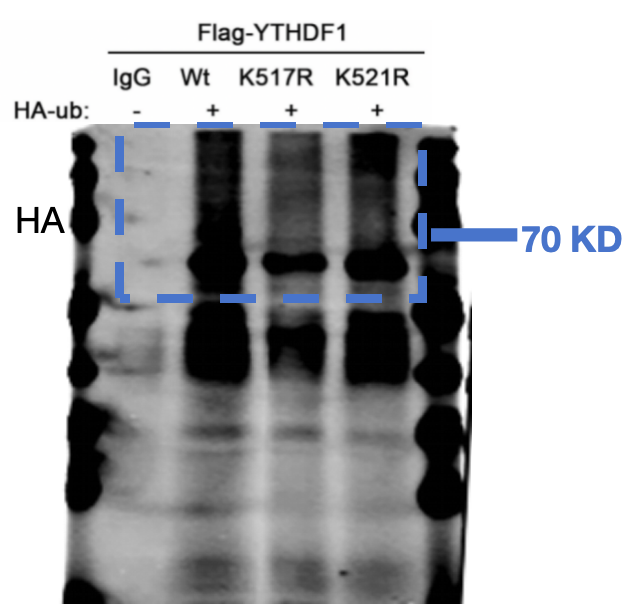

Supplement: Supplementary file 10 — Source data Fig. 4 [file 44319_2024_310_MOESM10_ESM.zip › FIgure 4/4G/Western co-IP_HA.tiff]

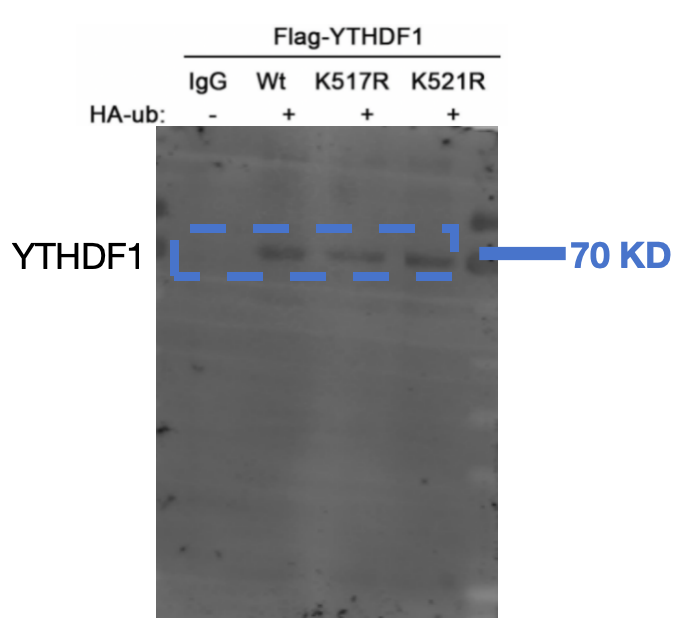

Supplement: Supplementary file 10 — Source data Fig. 4 [file 44319_2024_310_MOESM10_ESM.zip › FIgure 4/4G/Western IP_YTHDF1.tiff]

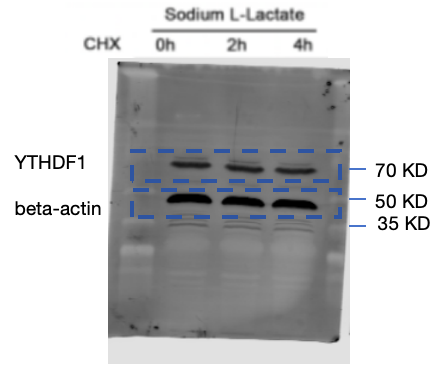

Supplement: Supplementary file 11 — Source data Fig. 5 [file 44319_2024_310_MOESM11_ESM.zip › Figure 5/5G/Western YTHDF1 beta-actin.tiff]

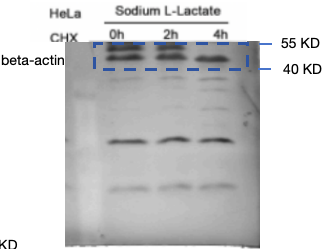

Supplement: Supplementary file 11 — Source data Fig. 5 [file 44319_2024_310_MOESM11_ESM.zip › Figure 5/5G/Western beta-actin.tiff]

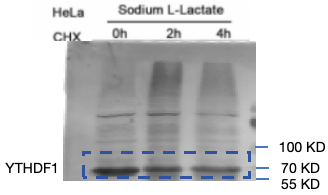

Supplement: Supplementary file 11 — Source data Fig. 5 [file 44319_2024_310_MOESM11_ESM.zip › Figure 5/5G/Western YTHDF1.tiff]

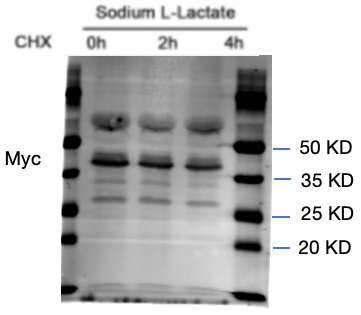

Supplement: Supplementary file 11 — Source data Fig. 5 [file 44319_2024_310_MOESM11_ESM.zip › Figure 5/5G/Western Myc.tiff]

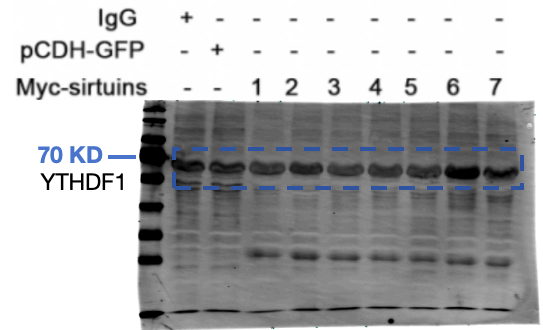

Supplement: Supplementary file 11 — Source data Fig. 5 [file 44319_2024_310_MOESM11_ESM.zip › Figure 5/5A/Western Input_YTHDF1.tiff]

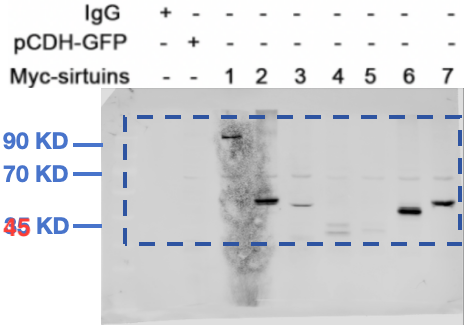

Supplement: Supplementary file 11 — Source data Fig. 5 [file 44319_2024_310_MOESM11_ESM.zip › Figure 5/5A/Western co- IP_Mycs.tiff]

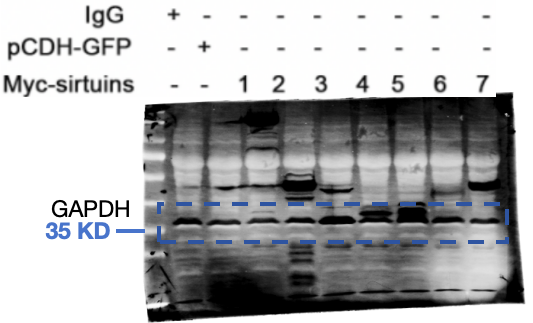

Supplement: Supplementary file 11 — Source data Fig. 5 [file 44319_2024_310_MOESM11_ESM.zip › Figure 5/5A/Western Input_GAPDH.tiff]

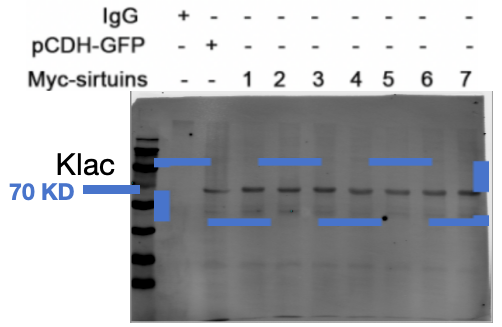

Supplement: Supplementary file 11 — Source data Fig. 5 [file 44319_2024_310_MOESM11_ESM.zip › Figure 5/5A/Western co-IP_Klac.tiff]

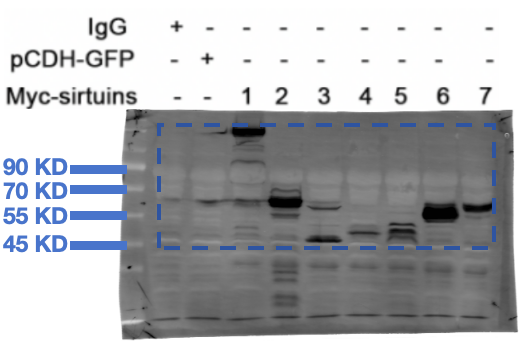

Supplement: Supplementary file 11 — Source data Fig. 5 [file 44319_2024_310_MOESM11_ESM.zip › Figure 5/5A/Western Input_Mycs.tiff]

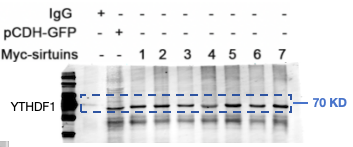

Supplement: Supplementary file 11 — Source data Fig. 5 [file 44319_2024_310_MOESM11_ESM.zip › Figure 5/5A/Western IP_YTHDF1.tiff]

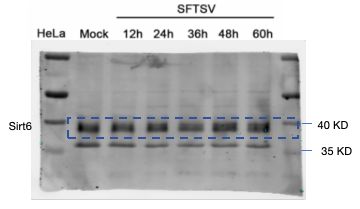

Supplement: Supplementary file 11 — Source data Fig. 5 [file 44319_2024_310_MOESM11_ESM.zip › Figure 5/5F /Western Sirt6.tiff]

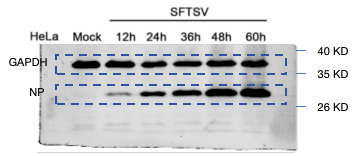

Supplement: Supplementary file 11 — Source data Fig. 5 [file 44319_2024_310_MOESM11_ESM.zip › Figure 5/5F /Western NP GAPDH.tiff]

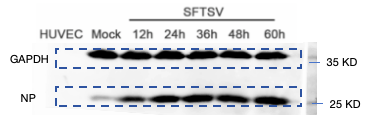

Supplement: Supplementary file 11 — Source data Fig. 5 [file 44319_2024_310_MOESM11_ESM.zip › Figure 5/5F /Western HUVEC- NP GAPDH.tiff]

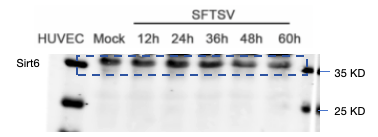

Supplement: Supplementary file 11 — Source data Fig. 5 [file 44319_2024_310_MOESM11_ESM.zip › Figure 5/5F /Western HUVEC-Sirt6.tiff]

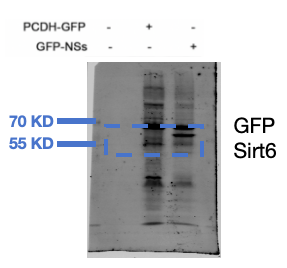

Supplement: Supplementary file 11 — Source data Fig. 5 [file 44319_2024_310_MOESM11_ESM.zip › Figure 5/5E /Western co-IP GFP Sirt6.tiff]

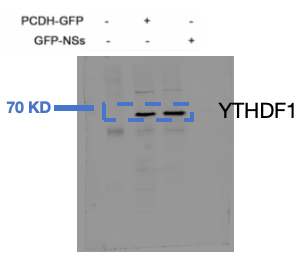

Supplement: Supplementary file 11 — Source data Fig. 5 [file 44319_2024_310_MOESM11_ESM.zip › Figure 5/5E /Western IP YTHDF1.tiff]

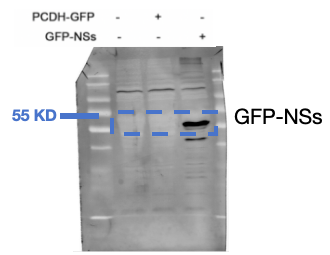

Supplement: Supplementary file 11 — Source data Fig. 5 [file 44319_2024_310_MOESM11_ESM.zip › Figure 5/5E /Western Input GFP-NSs.tiff]

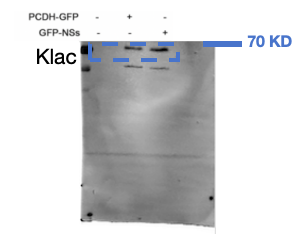

Supplement: Supplementary file 11 — Source data Fig. 5 [file 44319_2024_310_MOESM11_ESM.zip › Figure 5/5E /Western Klac.tiff]

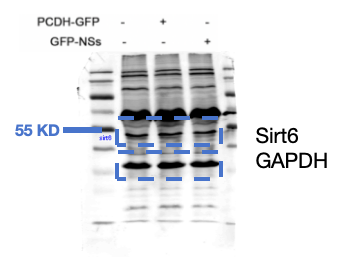

Supplement: Supplementary file 11 — Source data Fig. 5 [file 44319_2024_310_MOESM11_ESM.zip › Figure 5/5E /Western input sirt6 GAPDH.tiff]

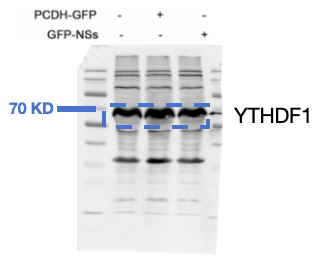

Supplement: Supplementary file 11 — Source data Fig. 5 [file 44319_2024_310_MOESM11_ESM.zip › Figure 5/5E /Western Input YTHDF1.tiff]

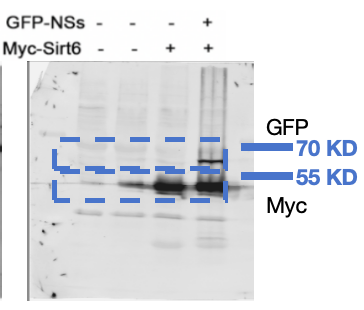

Supplement: Supplementary file 11 — Source data Fig. 5 [file 44319_2024_310_MOESM11_ESM.zip › Figure 5/5C/Western Input_GFP Myc.tiff]

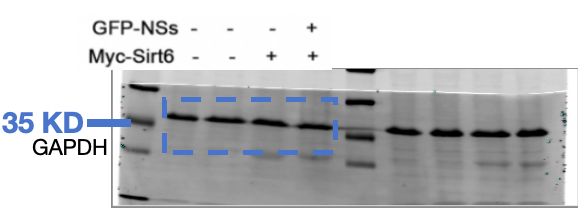

Supplement: Supplementary file 11 — Source data Fig. 5 [file 44319_2024_310_MOESM11_ESM.zip › Figure 5/5C/Western Input_GAPDH.tiff]

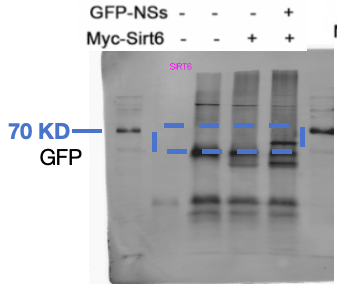

Supplement: Supplementary file 11 — Source data Fig. 5 [file 44319_2024_310_MOESM11_ESM.zip › Figure 5/5C/Western IP_GFP.tiff]

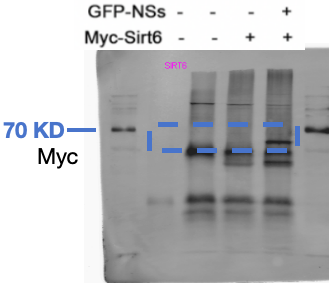

Supplement: Supplementary file 11 — Source data Fig. 5 [file 44319_2024_310_MOESM11_ESM.zip › Figure 5/5C/Western IB_Myc.tiff]

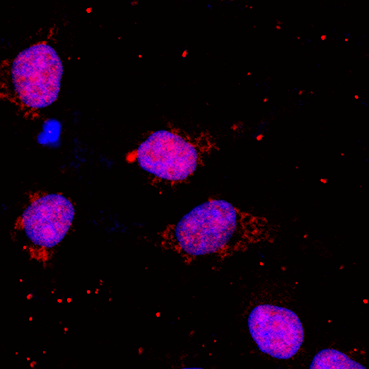

Supplement: Supplementary file 11 — Source data Fig. 5 [file 44319_2024_310_MOESM11_ESM.zip › Figure 5/5D/hela mock merge.tif]

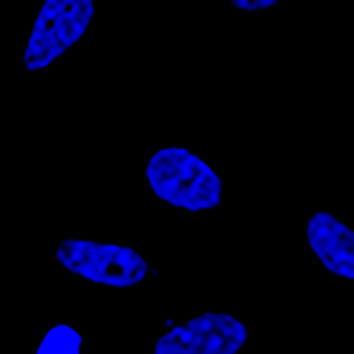

Supplement: Supplementary file 11 — Source data Fig. 5 [file 44319_2024_310_MOESM11_ESM.zip › Figure 5/5D/hela GFP-Nss overexpression nucleus.tif]

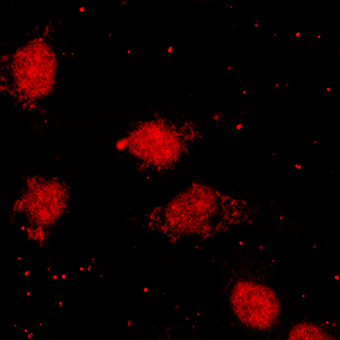

Supplement: Supplementary file 11 — Source data Fig. 5 [file 44319_2024_310_MOESM11_ESM.zip › Figure 5/5D/hela mock Sirt6.tif]

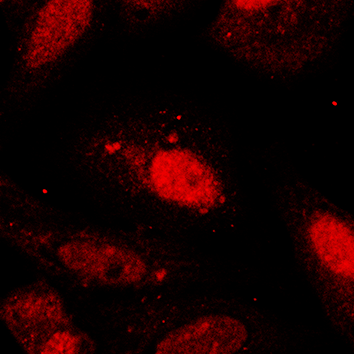

Supplement: Supplementary file 11 — Source data Fig. 5 [file 44319_2024_310_MOESM11_ESM.zip › Figure 5/5D/hela GFP-Nss overexpression Sirt6.tif]

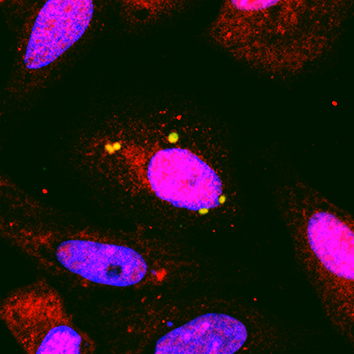

Supplement: Supplementary file 11 — Source data Fig. 5 [file 44319_2024_310_MOESM11_ESM.zip › Figure 5/5D/hela GFP-Nss overexpression Merge.tif]

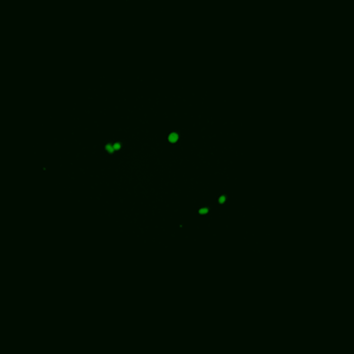

Supplement: Supplementary file 11 — Source data Fig. 5 [file 44319_2024_310_MOESM11_ESM.zip › Figure 5/5D/hela GFP-Nss overexpression GFP.tif]

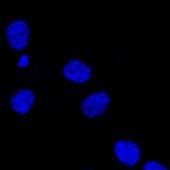

Supplement: Supplementary file 11 — Source data Fig. 5 [file 44319_2024_310_MOESM11_ESM.zip › Figure 5/5D/hela mock nucleus.tif]

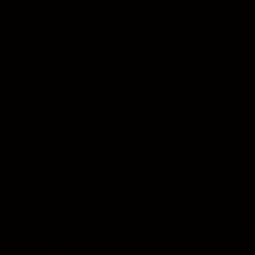

Supplement: Supplementary file 11 — Source data Fig. 5 [file 44319_2024_310_MOESM11_ESM.zip › Figure 5/5D/hela mock NSs.tif]

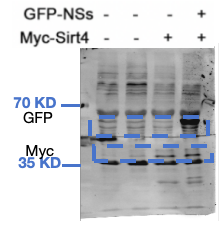

Supplement: Supplementary file 11 — Source data Fig. 5 [file 44319_2024_310_MOESM11_ESM.zip › Figure 5/5B/Western Input_GFP Myc.tiff]

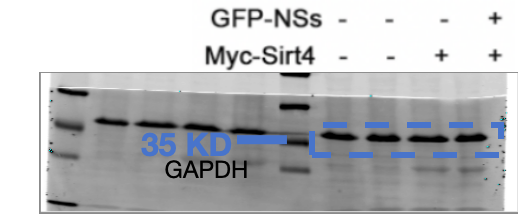

Supplement: Supplementary file 11 — Source data Fig. 5 [file 44319_2024_310_MOESM11_ESM.zip › Figure 5/5B/Western Input_GAPDH.tiff]

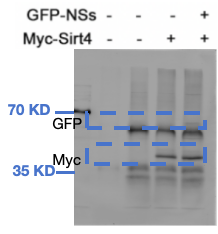

Supplement: Supplementary file 11 — Source data Fig. 5 [file 44319_2024_310_MOESM11_ESM.zip › Figure 5/5B/Western IP_GFP IB_Myc.tiff]
