# Supplementary material for: Severe fever with thrombocytopenia syndrome virus induces lactylation of m6A reader protein YTHDF1 to facilitate viral replication
Source: EMBO Rep. 2024 Nov 4;25(12):5599–619. doi: 10.1038/s44319-024-00310-7 (PMC11624280; doi:10.1038/s44319-024-00310-7)
Supplement: Supplementary file 13 — Expanded View Figures [file 44319_2024_310_MOESM13_ESM.pdf]

Expanded View Figures

Figure EV1. The regulatory relationship between SFTSV infection and m6A modification-related enzymes.

(**A**) Immunofluorescence assay was performed to measure the distribution of METTL3 (Green) in HeLa cells after SFTSV infection. Cell nuclei were stained with DAPI. Scale bar = 10 µm. (**B**) Immunofluorescence assay was performed to measure the distribution of METTL14 (Green) in HeLa cells after SFTSV infection. Cell nuclei were stained with DAPI. Scale bar = 10 µm. (**C**) Immunofluorescence assay was performed to measure the distribution of ALKBH5 (Red) in HeLa cells after SFTSV infection. Cell nuclei were stained with DAPI. Scale bar = 10 µm. (**D**) HeLa cells were transfected with siMETTL3 or siNC for 36 h, and then infected with SFTSV for 36 h. The expression levels were detected by western blot. GAPDH was used as a loading control. (**E**) HeLa cells were transfected with siALKBH5 or siNC for 36 h, and then infected with SFTSV for 36 h. The expression levels were detected by western blot. GAPDH was used as a loading control. (**F**) HeLa cells were transfected with PECMV-METTL3 plasmid for 36 h, and then infected with SFTSV for 36 h. The expression levels were detected by western blot. GAPDH was used as a loading control. (**G**) HeLa cells were transfected with PECMV-ALKBH5 plasmid for 36 h, and then infected with SFTSV for 36 h. The expression levels were detected by western blot. GAPDH was used as a loading control.

 © The Author(s)

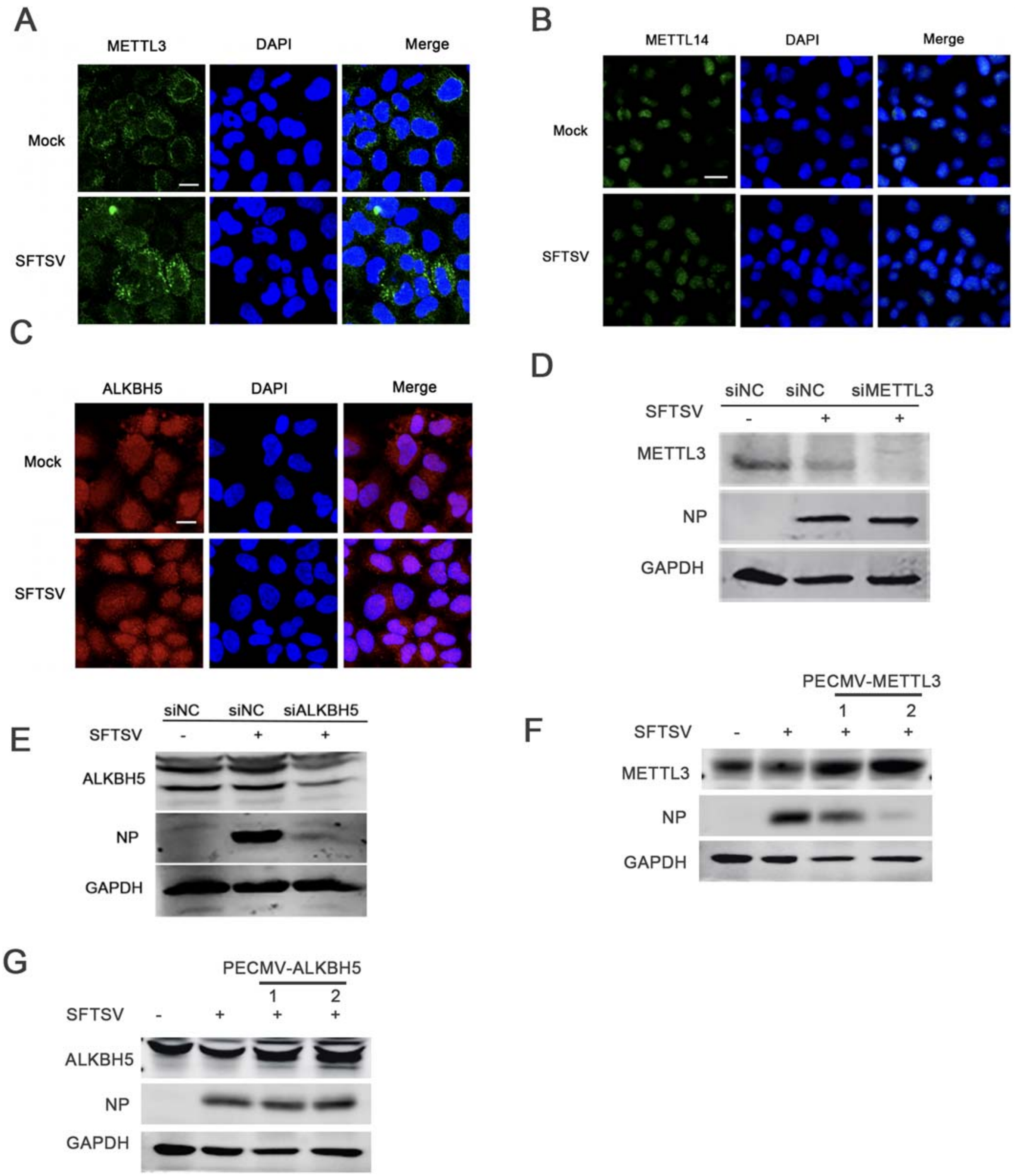
A
METTL3
DAPI
Merge
Mock
SFTSV
B
METTL14
DAPI
Merge
Mock
SFTSV
C
ALKBH5
DAPI
Merge
Mock
SFTSV
D
siNC siNC siMETTL3
SFTSV - + +
METTL3
NP
GAPDH
E
siNC siNC siALKBH5
SFTSV - + +
ALKBH5
NP
GAPDH
F
PECMV-METTL3
1 2
SFTSV - + + +
METTL3
NP
GAPDH
G
PECMV-ALKBH5
1 2
SFTSV - + + +
ALKBH5
NP
GAPDH

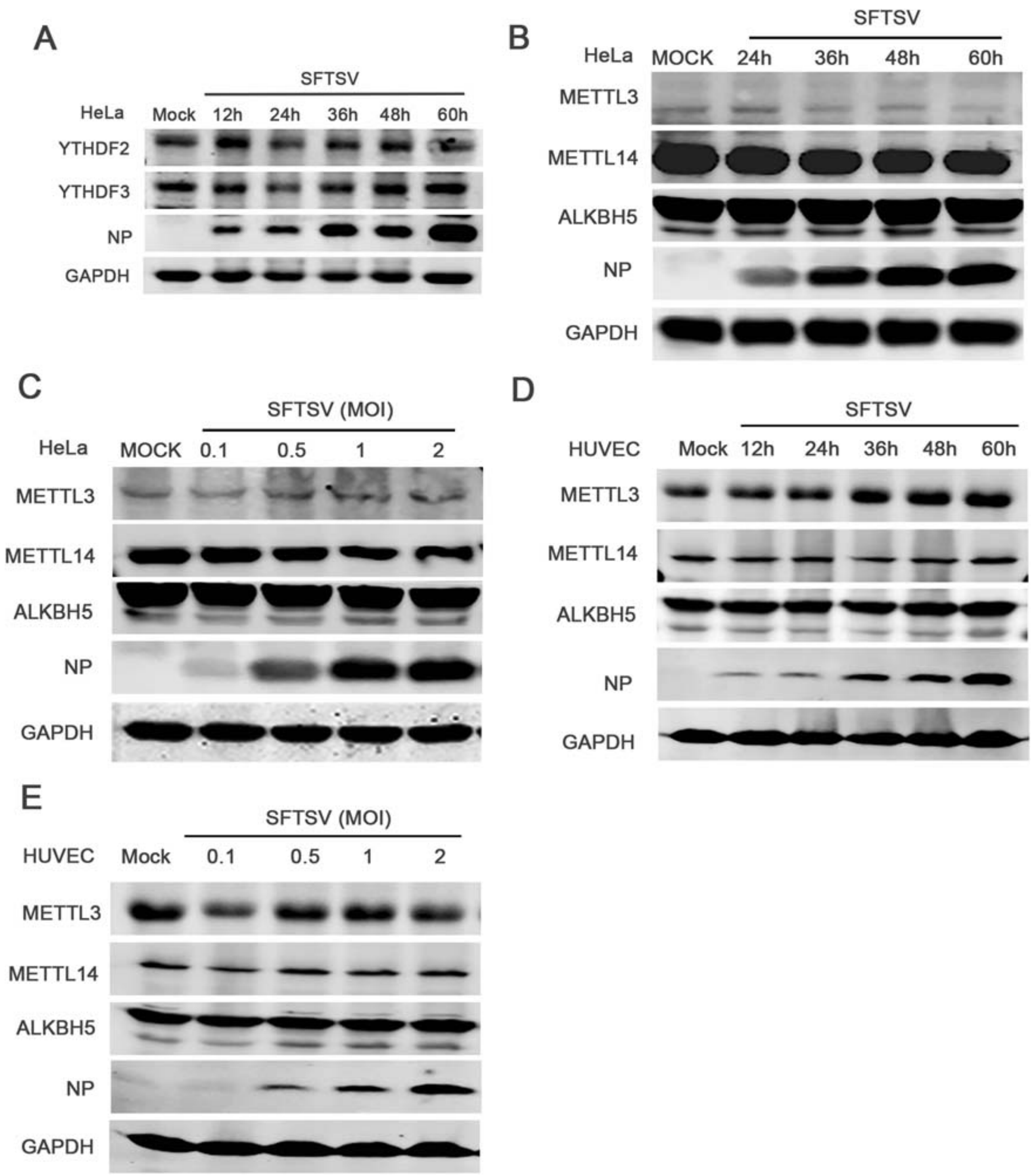

Figure EV2. The expression of m6A-related methyltransferases, demethylases, and reader proteins in HeLa and HUVEC cells after SFTSV infection.

(**A**) HeLa cells were infected with SFTSV (MOI = 1) in a time-dependent manner, and the expression level of YTHDF2 and YTHDF3 protein were detected by western blot. GAPDH was used as a loading control. (**B**) HeLa cells were infected with SFTSV in a time-dependent manner. The expression of ALKBH5, METTL3, and METTL14 were detected by western blot. (**C**) HeLa cells were infected with SFTSV in a dose-dependent manner. The expression of ALKBH5, METTL3, and METTL14 were detected by western blot. (**D**) HUVEC cells were infected with SFTSV in a time-dependent manner. The expression of ALKBH5, METTL3, and METTL14 were detected by western blot. (**E**) HUVEC cells were infected with SFTSV in a dose-dependent manner. The expression of ALKBH5, METTL3, and METTL14 were detected by western blot.

 © The Author(s)

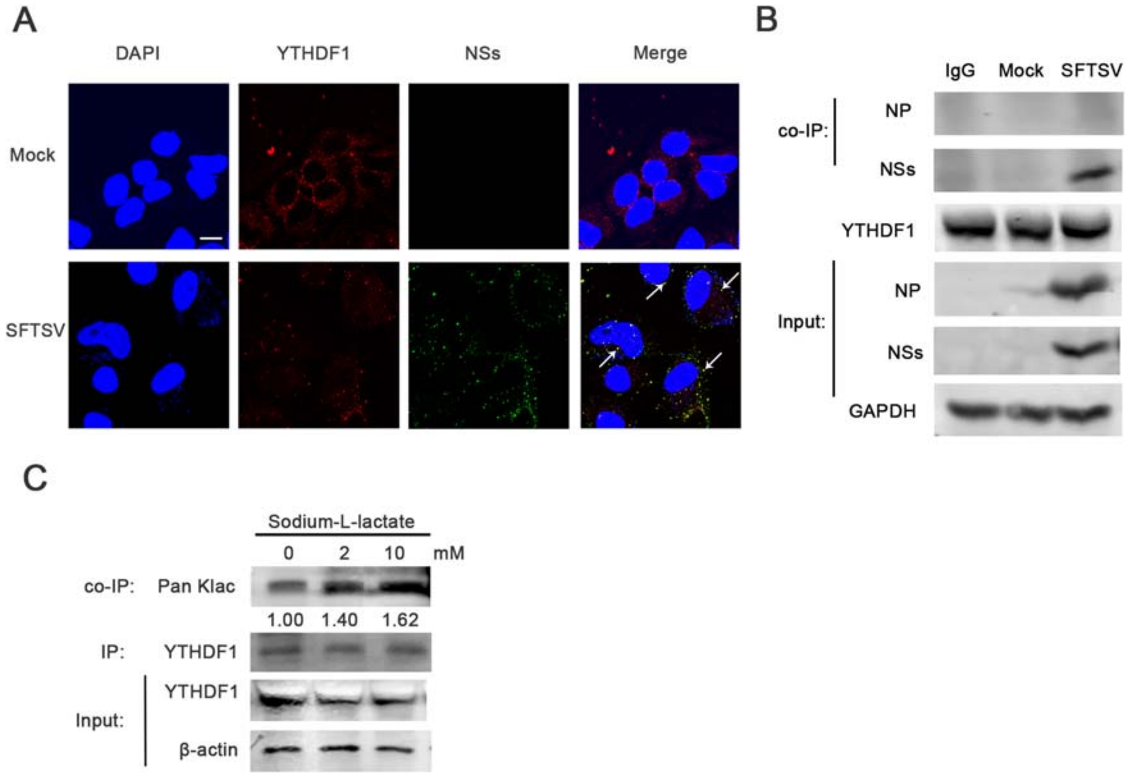

Figure EV3. The virulence factor NSs of SFTSV co-localized with YTHDF1.

Exogenously added Soudium-L-lactate could increase the lactylation modification on YTHDF1. (**A**) Immunofluorescence assay was used to measure the expression level of YTHDF1 (red) and the colocalization of YTHDF1 with NSs (green) in HeLa cells after SFTSV infection. Cell nucleus were stained with DAPI. Scale bar = 10 µm. (**B**) The interaction between endogenous YTHDF1 and NSs was detected by endogenous IP using a YTHDF1-specific antibody in uninfected or SFTSV-infected (MOI = 1) HeLa cells. Non-specific IgG antibody was used as negative control. (**C**) HeLa cells were treated with 2 mM or 10 mM Sodium L-Lactate for 12 h, and the whole cell lysates were collected by co-IP using YTHDF1-specific antibody. The lactylation level were detected by western blot using anti-Klac antibody.

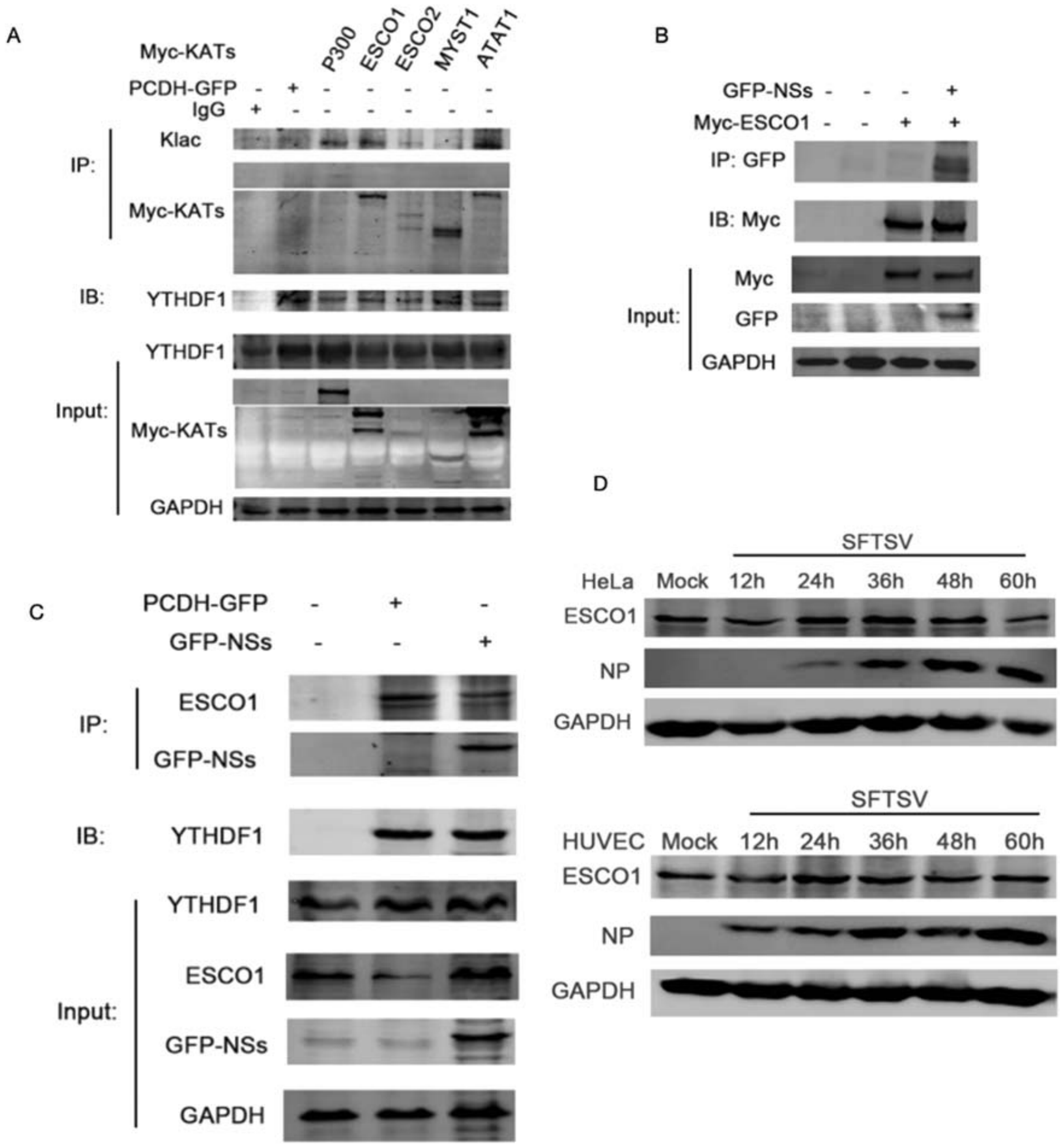

Figure EV4. Overexpression of SFTSV NSs increased the binding affinity of YTHDF1 and ESCO1.

(**A**) The interaction between endogenous YTHDF1 and Myc-P300, Myc-ESCO1, Myc-ESCO2, Myc-MYST1, and Myc-ATAT1 was detected by endogenous IP using a YTHDF1-specific antibody in HEK-293T cells. The lactylation level were detected by anti-Klac antibody. (**B**) The interaction between GFP-NSs and Myc-ESCO1 was detected by IP using a Myc-Specific antibody in HEK-293T cells. (**C**) IP was performed to measure the lactylation changes of YTHDF1 and the interaction between endogenous YTHDF1 and ESCO1 after overexpression of GFP-NSs by using YTHDF1-Specific antibody in HEK-293T cells. (**D**) The expression of ESCO1 protein was detected in HeLa and HUVEC cells in a time-dependent manner by western blot. GAPDH was used as a loading control.

 © The Author(s)

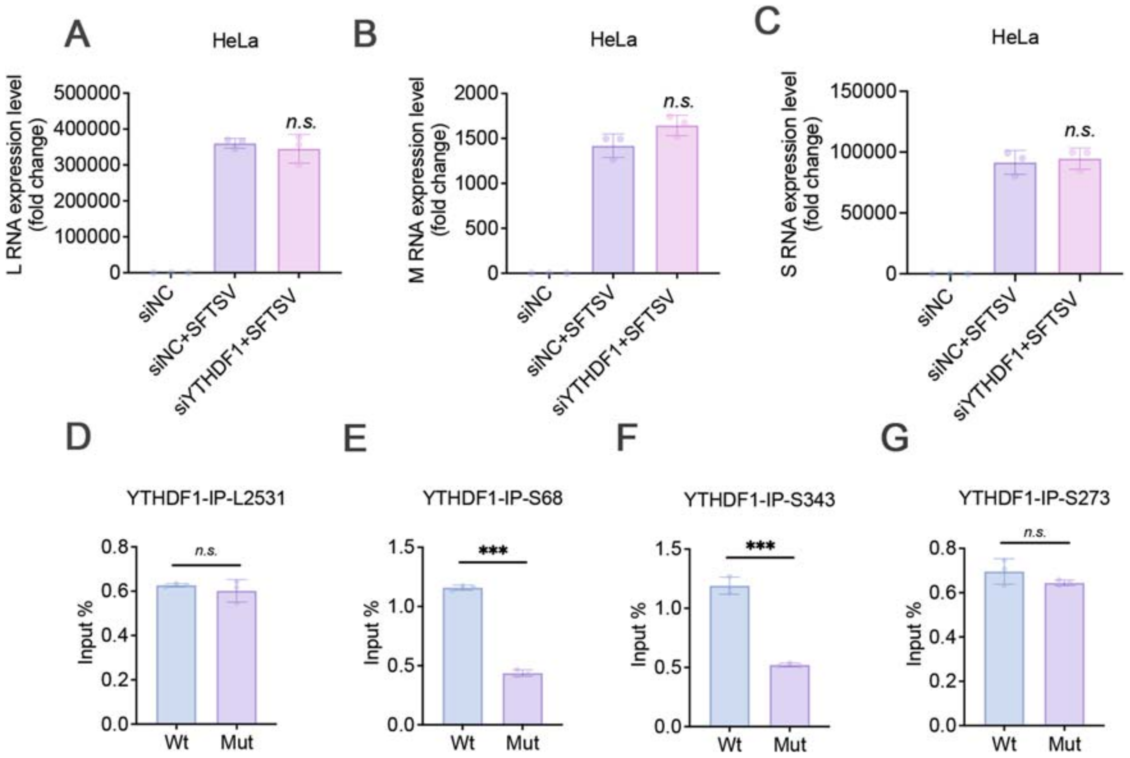

Figure EV5. The impact of knocking down YTHDF1 on SFTSV RNAs.

The effect of point mutations at the m6A sites on the binding of S RNA to YTHDF1. (**A–C**) HeLa cells were transfected with siNC (Negative control) or siYTHDF1 for 24 h, and then infected with SFTSV (MOI = 1). The expression level of L, M, and S RNA of SFTSV were detected at 36 h by qPCR. The fold enrichment was determined by calculating the 2-Δct of the sample relative to the GAPDH. The results are represented as the means ± SD of $n = 3$ biological replicates. Statistical significance was determined by a two-sided Student's *t* test (n.s. = 0.7023, 0.0872, 0.5662). (**D-G**) HEK-293T cells were transfected with Wild-type plasmids or site-mutant plasmids for 24 h. YTHDF1-IP-qRT-PCR were performed to collect m6A-modified RNAs. The fold enrichment was determined by calculating the 2-Δct of the sample relative to input. The results are represented as the mean ± SD of $n = 3$ biological replicates. Statistical significance was determined by a two-sided Student's *t* test (L2531: n.s. = 0.4370, S68: ***$P < 0.0001$, S343: ***$P < 0.0001$, S273: n.s. = 0.2055).

© The Author(s)
